# Identifying anomalous nuclear radioactive sources using Poisson kriging and mobile sensor networks

**DOI:** 10.1371/journal.pone.0216131

**Published:** 2019-05-01

**Authors:** Jifu Zhao, Zhe Zhang, Clair J. Sullivan

**Affiliations:** 1 Department of Nuclear, Plasma, and Radiological Engineering, University of Illinois at Urbana-Champaign, Urbana, Illinois, United States of America; 2 Cyberinfrastructure and Geospatial Information Laboratory, Department of Geography & Geographic Information Science, University of Illinois at Urbana-Champaign, Urbana, Illinois, United States of America; 3 Department of Geography, Texas A&M University, College Station, Texas, United States of America; The University of the South Pacific, FIJI

## Abstract

Nuclear security is a critical concept for public health, counter-terrorism efforts, and national security. Nuclear radioactive materials should be monitored and secured in near real-time to reduce potential danger of malicious usage. However, several challenges have arose to detect the anomalous radioactive source in a large geographical area. Radiation naturally occurs in the environment. Therefore, a non-zero level of radiation will always exist with or without an anomalous radioactive source present. Additionally, radiation data contain high levels of uncertainty, meaning that the measured radiation value is significantly affected by the velocity of the detector and background noise. In this article, we propose an innovative approach to detect anomalous radiation source using mobile sensor networks combined with a Poisson kriging technique. We validate our results using several experiments with simulated radioactive sources. As results, the accuracy of the model is extremely high when the source intensity is high or the anomalous source is close enough to the detector.

## Introduction

Nuclear weapons, bombs, as well as radiological dispersal devices are threats to national security and human health. However, detecting anomalous radioactive sources over a large geographical area has several challenges. First, radiation naturally occurs in the ground, building materials, and cosmic rays. Therefore, a non-zero level of radiation will always exist, which presents the problem of detecting a radioactive source with a low signal-to-noise ratio (SNR). Here, the radiation source is the anomalous radiation signal and the background radiation is the noise.

Additionally, radiation data contain high levels of uncertainty. The GPS location of the detector is only accurate within 1 to 3 meters, and the measured radiation value is significantly affected by the velocity of the detector, background noise, shielding materials, weather conditions, and the distance to the radiation source. For instance, SNR decreases dramatically as the distance from the radiation detector to actual sources increases. For this investigation, two types of radiations are considered: naturally occurring background radiation and anomalous radioactive sources (e.g., ^137^*Cs* and ^60^*Co*). Examples of natural background radiation are the radiation emitted from soil, rock, and buildings. Anomalous radioactive sources include nuclear weapons, dirty bombs, or the precursors to such weapons.

Various types of methods have been developed to estimate background radiation and detect radioactive sources. Reinhart [1] developed an integrated system for gamma-ray spectral mapping and anomaly detection. A temporal anomaly detection algorithm has been developed to perform source injection simulation. The kriging method was used to interpolate the radiation level of the areas that have missing data. Tansey et al. [2] presented a method called multi-scale spatial density smoothing based on a recursive dyadic partition of the sample space and shared much in common with other multi-scale methods, such as wavelets and Polya-tree priors. Heuvelink and Griffith [3] introduced a space-time kriging approach to characterize the variability of radiation data using the monthly averaged gamma dose rates collected during a 5-year period. In this study, the space-time variable of interest was treated as a sum of independent stationary spatio-temporal components, which led to a sum-metric space-time variogram model. Various methods based on maximum likelihood estimation (MLE) have been proposed under different circumstances. For example, Morelande et al. [4] analyzed the estimation problem for multiple radioactive sources using a MLE method. Zhao et al. [5] implemented MLE with grid search to estimate source intensity and location. Zheng et al. [6] developed methods based on MLE to estimate the spatial and temporal distribution of background radiation using mobile sensor networks. Additionally, other methods, such as Bayesian methods [7–9], mean of estimators and mean of measurements [10], Markov Chain Monte Carlo sampling [11], and least squares estimation [12] were also proposed. However, the approaches mentioned above mostly used the predefined structure of sensor networks to calculate the background radiation levels. The fixed radiation sensor networks such as traditional radiation portal monitors often suffer from the inverse square law of the radiation, whereby the source intensity falls off as 1/*r*^2^ when the distance between detector and radioactive source increases. This problem becomes more challenging when the source is moving across space and time. Therefore, the mobile detector sensor network should be introduced, so that the detector can be placed closer to the moving source [5, 13–15].

Jeong et al. [16] developed structural similarities of the surface networks to identify radiation level changes. The structural similarities over time provided moderate correlations for the radiation level changes, and the results indicated that there were statistically significant differences between each month’s radiation levels. However, it is difficult to understand whether this difference comes from background fluctuations or radioactive materials. Therefore, more research needs to be done to analyze the correlation between background radiation and other uncertain factors. The geographically weighed correlation method has been applied to a small number of radiation data sets to observe the correlation between radiation level measures and detectors’ velocities [17]. However, the *t* value of the model was missing and there was no justification of the statistical significance of the results. Furthermore, other factors such as weather condition variables were not considered. Jeong et al. [18] developed a similarity measuring approach where cosine, Jaccard, and Euclidean distance similarity measures were used in anomalous radiation detection. They assumed that the presence of an anomalous source should be suspected if a radiation measure differed from the background. The similarity (or dissimilarity) measures between two independent measurements were represented as two sets of vectors. However, the overall similarity measures did not present high correlations between 2013 and 2014 and the accuracy of the similarity measure was not discussed in the research. Later, various types of spatial algorithms such as the Getis-Ord local statistic (G*), a novel robust version of the Getis-Ord local statistic (r-G*), and a traditional Local Indicators of Spatial Association (LISA) approach, have been applied to radiation data [19]. Results were compared by using receiver operating characteristic (ROC) curves over traditional approaches.

Kriging is a geostatistical interpolation method that uses the given measurements to estimate measurements at positions where data were not collected [20]. There are several commonly used kriging models, including ordinary kriging, simple kriging, and universal kriging. For radiation detection, all measurements are integers, and the signals are assumed to follow the Poisson distribution of the expected radiation level. In this case, the often-used Gaussian assumption is not suitable for modeling radiation data because the radiation count for certain detectors could have relatively low mean values. Also, for radiation data, it is more important to estimate the radiation level, which can be represented as the average radiation count rate, then simply interpolating using measured data. Therefore, it is more reasonable to use the Poisson kriging approach [1]. Monestiez et al. [21] proposed the Poisson kriging to model the spatial distribution of Balaenoptera physalus (a kind of whale) using the sparse count data. McShane et al. [22] developed a similar model to analyze the spatially correlated count data. Recently, Bellier et al. [23] extended Poisson kriging to nonstationary hierarchical model for count data. Since then, Poisson kriging has been applied to count data for different areas, including cholera and dysentery incidence [24], cancer [25, 26], and wildlife population [21, 23].

This study employed the Poisson kriging method to estimate nuclear radiation distribution and identify anomalous radioactive sources using data collected by mobile sensor networks. The performance of the proposed algorithm is demonstrated using the experimental data with simulated radioactive sources.

## Background and methodology

### Radiation transport model

There are two types of radiations involved in this study. The background radiation (*b*) and radiation comes from radioactive sources (*s*). Nuclear radiation can be measured by various detectors, which are in integer format (counts per second, or cps). The measured radiation count rate is assumed to follow the Poisson distribution [27]. The probability of a detector collects *m* counts in a unit time with expectation λ is expressed as:

$$\begin{array}{c} p\left(x=m\right)=\frac{\lambda^{m}}{m!}\cdot e^{-\lambda }, \end{array}$$ (1)

where λ represents the average radiation count rate at a given position. Variable λ is in the form of λ = *b* + *s*. Variable *s* is equal to zero when there are no radioactive sources present, and it is mainly influenced by the shielding materials and distance between the actual source and detector. Variable *b* is influenced by many factors, such as the surrounding buildings and weather condition. Typically, *b* is the function of location ***r*** and time *t*. When the time interval considered is not long, *b* can be assumed to be constant around location ***r***.

### Poisson kriging and semi-variogram

Kriging is an interpolation method that is used to predict spatial attributes at unknown times and locations [28]. Poisson kriging was originally developed based on the population weighted semi-variogram estimators and used to analyze the count data [21]. Compared with ordinary kriging, Poisson kriging assumes the data follows Poisson distribution. The semi-variogram *γ*(***r***_*i*_, ***r***_*j*_) was first defined by Matheron [29] as half the average squared difference between points ***r***_*i*_ and ***r***_*j*_ separated at distance *h*.

$$\begin{array}{c} \gamma \left(r_{i},r_{j}\right)=\frac{1}{2}Var\left(X\left(r_{i}\right)-X\left(r_{j}\right)\right), \end{array}$$ (2)

where *X*(⋅) is the measured value and *Var*(⋅) is the variance.

For radiation measurements, let random observation *X*_***r***_ be the count rate measurement at location ***r***. There exists some underlying distribution *Y*_***r***_ which represents the expected value of *X*_***r***_. In other words, *Y*_***r***_ represents λ in Eq 1 at position ***r***. The goal of Poisson kriging is to estimate the distribution of *Y* instead of simply interpolating *X*. Given latent variable *Y*_***r***_, measured radiation count rate *X*_***r***_ is assumed to follow the Poisson distribution:

$$\begin{array}{c} X_{r}|Y_{r}∼Poisson\left(Y_{r}\right). \end{array}$$ (3)

Further, *Y*_***r***_ is assumed to be a positive random field honoring order two stationarity [21], which has the mean *μ*_***r***_ and variance $\sigma_{r}^{2}$. To simplify the problem, *μ*_***r***_ and $\sigma_{r}^{2}$ are assumed to be constant (For the case of using a non-constant mean, the trend can be estimated first and the problem is still solvable [23]). Then we get:

$$\begin{array}{c} E[Y_{r}]=\mu \end{array}$$ (4)

$$\begin{array}{c} E\left[Y_{r}^{2}\right]=\mu^{2}+\sigma^{2} \end{array}$$ (5)

$$\begin{array}{c} E\left[X_{r}X_{r^{\prime }}|Y\right]=\delta_{r,r^{\prime }}Y_{r}+Y_{r}Y_{r^{\prime }}, \end{array}$$ (6)

where *δ*_***r***, ***r***′_ is the delta function (1 if ***r*** = ***r***′ and 0 otherwise).

For Poisson kriging, considering two locations ***r*** and ***r***′, *X*_***r***_ is assumed to not interact with *X*_***r***′_ directly, and *Y*_***r***_ are connected with *Y*_***r*′**_ only through their covariance. The covariance function *C*_*Y*_(***r***, ***r***′) = *Cov*(*Y*_***r***_, *Y*_***r*′**_) for *Y* is assumed to depend only on the distance ||***r*** − ***r***′|| between two locations:

$$\begin{array}{c} C_{Y}\left(r,r^{\prime }\right)=E\left[\left(Y_{r}-\mu \right)\left(Y_{r^{\prime }}-\mu \right)\right]=E\left[Y_{r}Y_{r^{\prime }}\right]-\mu^{2}. \end{array}$$ (7)

Traditionally, it is more common to use semi-variogram instead of covariance to model the correlation. The semi-variogram function for *X* is defined as $\gamma_{X}\left(r,r^{\prime }\right)=\frac{1}{2}E\left[{\left(X_{r}-X_{r^{\prime }}\right)}^{2}\right]$. Thus, the semi-variogram function for *Y* can be calculate as:

$$\begin{array}{c} \gamma_{Y}\left(r,r^{\prime }\right)=\gamma_{X}\left(r,r^{\prime }\right)+\delta_{r,r^{\prime }}\mu -\mu . \end{array}$$ (8)

From Eq 8, the covariance function and semi-variogram function of *Y* can be estimated using the formula:

$$\begin{array}{c} C_{Y}\left(r,r^{\prime }\right)=\sigma^{2}-\gamma_{Y}\left(r,r^{\prime }\right). \end{array}$$ (9)

For radiation detection with multiple detectors, suppose that there are *n* measurements *X*_1_, *X*_2_, ⋯, *X*_*n*_ from different locations ***r***_1_, ***r***_2_, ⋯, ***r***_*n*_, the estimate of *Y*_0_ for unmeasured location ***r***_0_ is assumed to be the linear combination of available measurements *X*_1_, *X*_2_, ⋯, *X*_*n*_:

$$\begin{array}{c} {\hat{Y}}_{0}=\sum_{i=1}^{n}\lambda_{i}X_{i}. \end{array}$$ (10)

Then the problem becomes finding λ_1_, λ_2_, ⋯, λ_*n*_ such that Eq 10 works as the optimal estimator so that the estimator ${\hat{Y}}_{0}$ is unbiased and the squared estimation error ${\left({\hat{Y}}_{0}-Y_{0}\right)}^{2}$ is minimized. The following *n* + 1 equations can be derived:

$$\begin{array}{c} \sum_{i=1}^{n}\lambda_{i}=1 \end{array}$$ (11)

$$\begin{array}{c} \sum_{j=1}^{n}\lambda_{j}C_{Y}\left(i,j\right)+\lambda_{i}\mu +\alpha =C_{Y}\left(i,0\right)\;for\;i=1,2,\cdots ,n, \end{array}$$ (12)

where *α* is the Lagrange multiplier.

Eqs 11 and 12 can be written in matrix format *A****x*** = ***b***. *A* is a (*n* + 1) by (*n* + 1) matrix, and ***x*** and ***b*** are both column vectors with (*n* + 1) elements, which are shown below:

$$\begin{array}{c} A=(\begin{array}{ccccc} C_{11}+\mu & C_{12} & \cdots & C_{1n} & 1\\ C_{21} & C_{22}+\mu & \cdots & C_{2n} & 1\\ C_{31} & C_{32} & \cdots & C_{3n} & 1\\ \vdots & \vdots & \ddots & \vdots & \vdots \\ C_{n1} & C_{n2} & \cdots & C_{nn}+\mu & 1\\ 1 & 1 & \cdots & 1 & 0 \end{array}) \end{array}$$ (13)

$$\begin{array}{c} x={\left(\lambda_{1},\lambda_{2},\lambda_{3},\cdots ,\lambda_{n},\alpha \right)}^{T} \end{array}$$ (14)

$$\begin{array}{c} b={\left(C_{10},C_{20},C_{30},\cdots ,C_{n0},1\right)}^{T}, \end{array}$$ (15)

where for simplicity, *C*_*Y*_ (*i*, *j*) is denoted by *C*_*ij*_.

The key to solving the Poisson kriging problem is to calculate the covariance function *C*_*ij*_. Since *C*_*ij*_ = *σ*^2^ − *γ*_*Y*_ (*i*, *j*), the semi-variogram function *γ*_*Y*_ (*i*, *j*) can be first estimated in order to estimate *C*_*ij*_. Semi-variogram function *γ*_*Y*_ (*i*, *j*) is assumed to be determined by the distance *h* = ||***r***_*i*_ − ***r***_*j*_|| only. Based on Eq 8, the semi-variogram function of *Y* can be calculate from the semi-variogram of *X*. Here, Eq 16 is used to estimate *γ*_*X*_ (*h*).

$$\begin{array}{c} {\hat{\gamma }}_{X}\left(h\right)=\frac{1}{N(h)}\sum_{i,j}{\left[\frac{1}{2}{\left(X_{i}-X_{j}\right)}^{2}\right]}_{I_{d_{ij}\approx h}}, \end{array}$$ (16)

where *N*(*h*) = ∑_*i*,*j*_ *I*_*d*_*ij*_ ≈ *h*_. *I*_*d*_*ij*_ ≈ *h*_ is the indicator function which is 1 when the distance between ***r***_*i*_ and ***r***_*j*_ is roughly equal to h. Otherwise, this indicator function is equal to 0. From Eq 8, the semi-variogram function *γ*_*Y*_ (*h*) can be easily inferred.

Eq 16 gives discrete estimates of ${\hat{\gamma }}_{X}\left(h\right)$. To get a continuous estimate, a semi-variogram model can be fitted based on the calculated ${\hat{\gamma }}_{X}\left(h\right)$ using nugget model, spherical model, exponential model, Gaussian model and so on. In this research work, we use exponential model and the equation is illustrated below:

$$\begin{array}{c} {\hat{\gamma }}_{X}\left(h\right)=c_{0}+c(1-exp(-\frac{h}{a})), \end{array}$$ (17)

where parameters *c*_0_, *c*, and *a* are determined using the measured data.

## Experiments and data

In this research work, two types of data were generated: background radiation data and radioactive source data. Background radiation data were measured using radiation detectors and radioactive source data were simulated using GADRAS (Gamma Detector Response and Analysis Software, developed and maintained by Sandia National Laboratories [30, 31]).

The radiation detector used in this research work is called Discreet Dual Detector (D3S detector) [32], which is designed to detect gamma-ray. D3S detector is a thallium activated cesium iodide (*CsI*(*Tl*)) scintillation detector. The dimension of the D3S detector is 132*mm* × 80*mm* × 23.5*mm* and its weight is 237 g. It has a 1,450 mAh Lithium polymer battery, which can last around 12 hours. The D3S detector has the gamma-ray detection range from 30 keV to 3 MeV, and it has 7% resolution at 662 keV. The sensitivity of the detector is 550 *cps*/*μSv*/*h* for ^137^*Cs* and the maximum throughput for gamma channel is 10,000 cps. D3S detector can collect radiation data and then transmit the data wirelessly to other devices through Bluetooth. In addition to radiation detector, a smart phone (e.g., Samsung Galaxy S6) was used to collect measured data from D3S detector as well as recording information such as GPS information and corresponding timestamps. In this work, a mobile software application was developed by the research group [6, 33] to connect the smart phone to the Amazon cloud, which enables near-real time data collection and processing. For example, the data is collected every second and transmitted into the cloud with some latency (e.g., around 10 minutes using current application. But this can be improved in the future).

**Fig 1:**
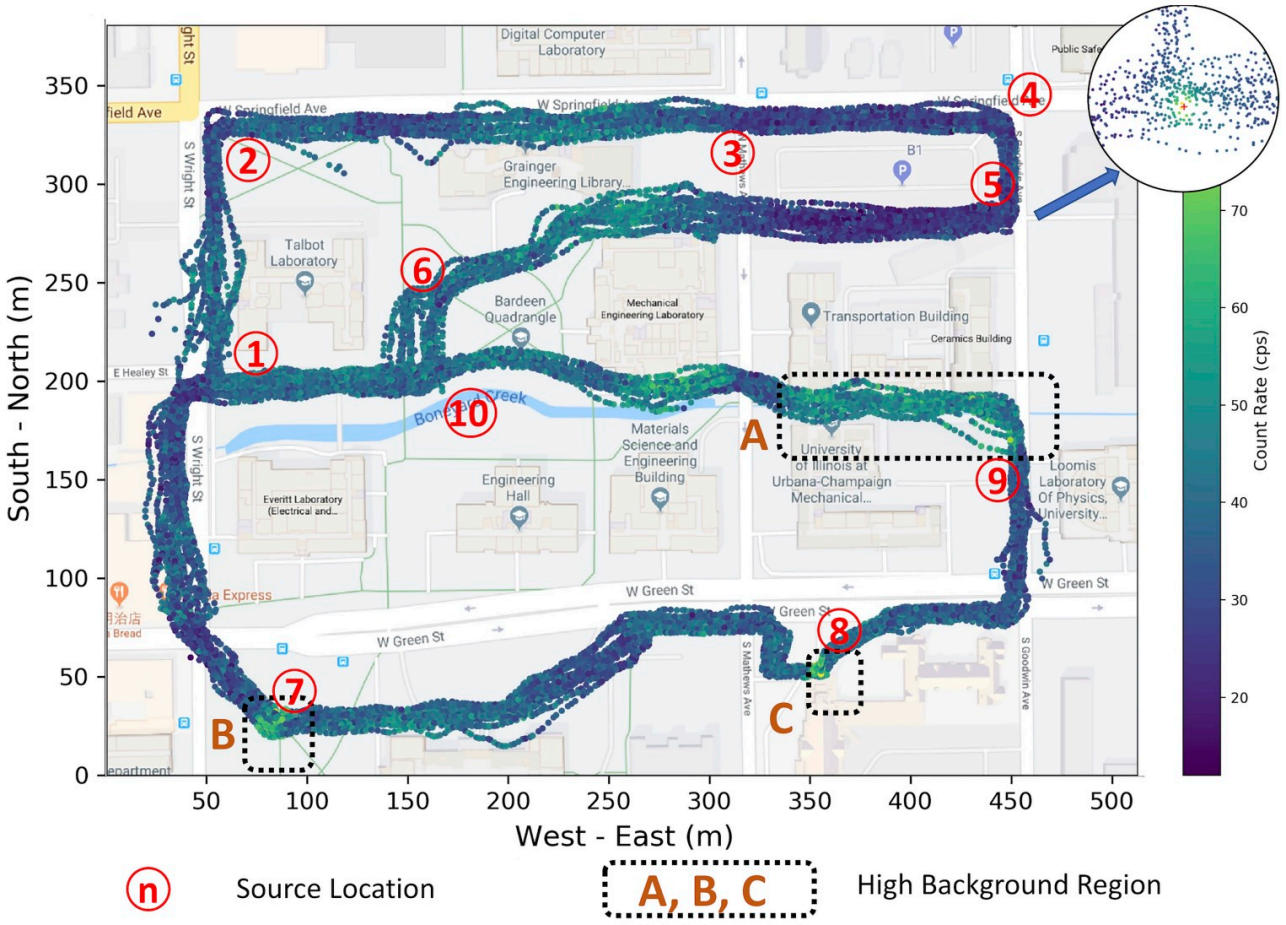
Illustration of experimental area, background radiation measurements, and radioactive source locations. Three relatively high background regions, namely the Nuclear Radiation Laboratory (A), the Alma Mater statue (B), and the Church (C) are denoted using dotted rectangles.

The experiment was conducted at the engineering campus of the University of Illinois at Urbana-Champaign. The entire experimental region is around 500 × 400 *m*^2^ large. The experiment was conducted on December 8th, 2017. The data were collected in the morning from 9:30 to 12:00 and in the afternoon from 14:30 to 15:30, and there was no actual radioactive source placed in the experimental region due to safety concerns. During the experiment, volunteers were required to put the D3S detectors in their pockets and walked in the normal walking speed (around 1.4 m/s) along the designated paths. The figure shows the experimental area and walking paths is published elsewhere [5] and redrawn here as shown in Fig 1. The figure also shows the measured background radiation data. Clearly, background radiation level gets increasing when the detector approaches buildings. Three typical high background radiation regions are denoted using dotted rectangles A, B, and C. The background radiation level is lower when the detector is approaching other places, such as grasslands and parking lots. The recorded data include the following attributes: detector’s ID, latitude and longitude of the position, radiation count rate (cps), and the corresponding timestamps. The measured data consists of 21, 883 data points in total.

For the radioactive source data, GADRAS was used to simulate the influence of radioactive sources. In the GADRAS simulation, a radioactive source is placed on the ground and a detector is placed 1 meter above the ground, which is consistent with the fact that detectors were put inside volunteers’ pockets during the experiment. In this work, ^137^*Cs* was chosen as the simulated radioactive source and GADRAS simulated the radiation count rate for D3S detector at multiple different distances. The source intensities varied from 100 *μCi* to 2,000 *μCi*. The information on source intensity and its corresponding radiation count rate (cps) measured when the detector is 1 meter away from the source is shown in Table 1.

**Table 1:** Simulated source intensity in counts per second.

Source Intensity	D3S Count Rate
100 *μCi* (3.7 × 10^6^ Bq)	127 cps
300 *μCi* (1.11 × 10^7^ Bq)	380 cps
500 *μCi* (1.85 × 10^7^ Bq)	626 cps
1,000 *μCi* (3.7 × 10^7^ Bq)	1,220 cps
2,000 *μCi* (7.4 × 10^7^ Bq)	2,354 cps

After the simulation, the sources were put into the originally measured background radiation data. For instance, we put the sources into the experimental region at 10 different randomly selected locations, which were denoted by numbers from 1 through 10 in Fig 1, covering the high background area and low background area. For each experiment, we injected one simulated radioactive source at the corresponding location. To test the performance of the proposed algorithm under different cases, the simulated radioactive source were put at 3 different distance (1 m, 5 m, and 10 m) from the walking paths. So, for each source location, there were 15 different scenarios in total (5 different source intensities and 3 different distances). The detailed locations of the injected radioactive sources are listed in Table 2.

**Table 2:** Locations of injected radioactive sources.

Source No.	Distance to Walking Paths		
	1 m	5 m	10 m
1	55.87, 196.93	59.95, 200.48	64.97, 205.15
2	54.77, 328.80	58.59, 324.80	63.70, 320.02
3	310.66, 330.58	306.74, 326.58	301.81, 321.24
4	446.81, 331.36	442.98, 328.36	441.54, 323.47
5	450.64, 281.66	447.15, 285.66	442.30, 290.77
6	172.72, 245.41	176.12, 243.41	180.37, 241.52
7	86.49, 23.57	86.49, 27.69	86.57, 32.69
8	360.92, 50.48	358.12, 53.26	354.88, 56.82
9	453.70, 179.14	450.38, 176.58	446.47, 173.80
10	167.96, 204.49	171.44, 206.16	175.36, 209.16

Source locations are shown with two numbers, meaning the location (in meters) in West-East direction and South-North direction corresponding to Fig 1.

**Fig 2:**
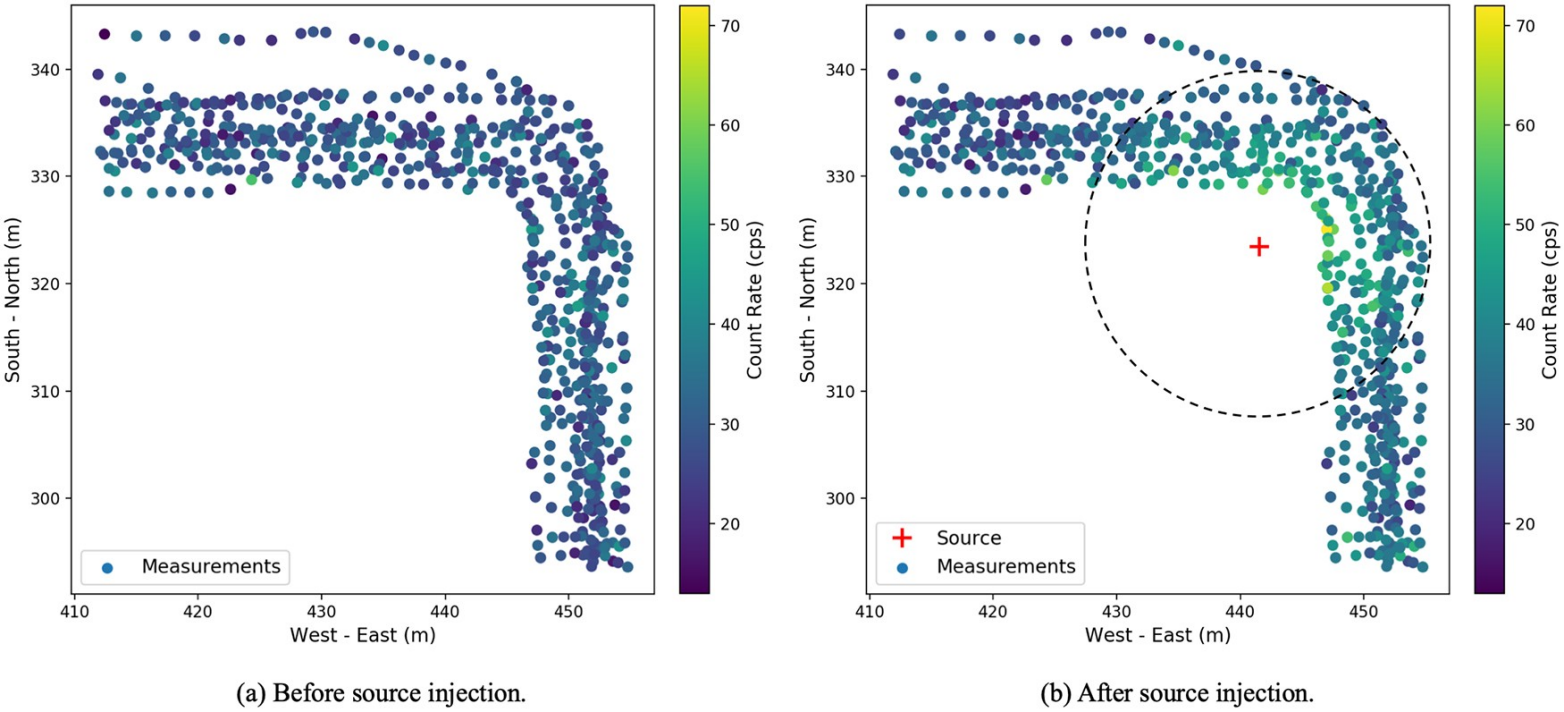
Illustration of synthetic source injection.

We used the data that were collected during the afternoon to inject radioactive sources. Fig 2 shows the measured count rate within a certain distance from the source has increased significantly. The radiation level of the data that is far away from the source remains the same.

## Results and discussion

### Nuclear radiation estimation

**Fig 3:**
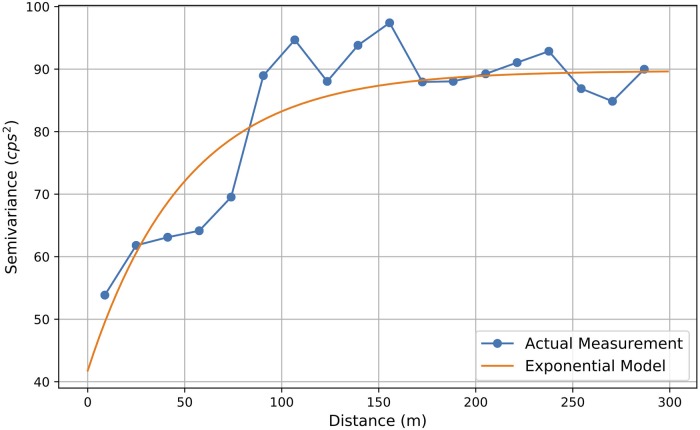
Semi-variogram plot of background radiation measurements.

According to Tobler’s first law, “*everything is related to everything else, but near things are more related than distant things*” [34]. Our experiments indicate that the data follows Tobler’s first law well. For instance, Fig 3 illustrates the semi-variogram of the collected background radiation data, where the x-axis represents the distance between radiation data points, and the y-axis represents the semi-variogram value. Using the background radiation data collected in the morning as shown in Fig 1, a series of discrete estimates of semi-variogram values are obtained, then an exponential model (Eq 17) can be fitted.

Fig 3 shows that the semi-variogram value increases with small data distances, which indicates strong correlation between radiation measurements. As the distance increases, the correlations decreases and the fitted line tends to saturate. This is also consistent with the expectation that as the distance becomes large enough, there is almost no correlation between two locations.

**Fig 4:**
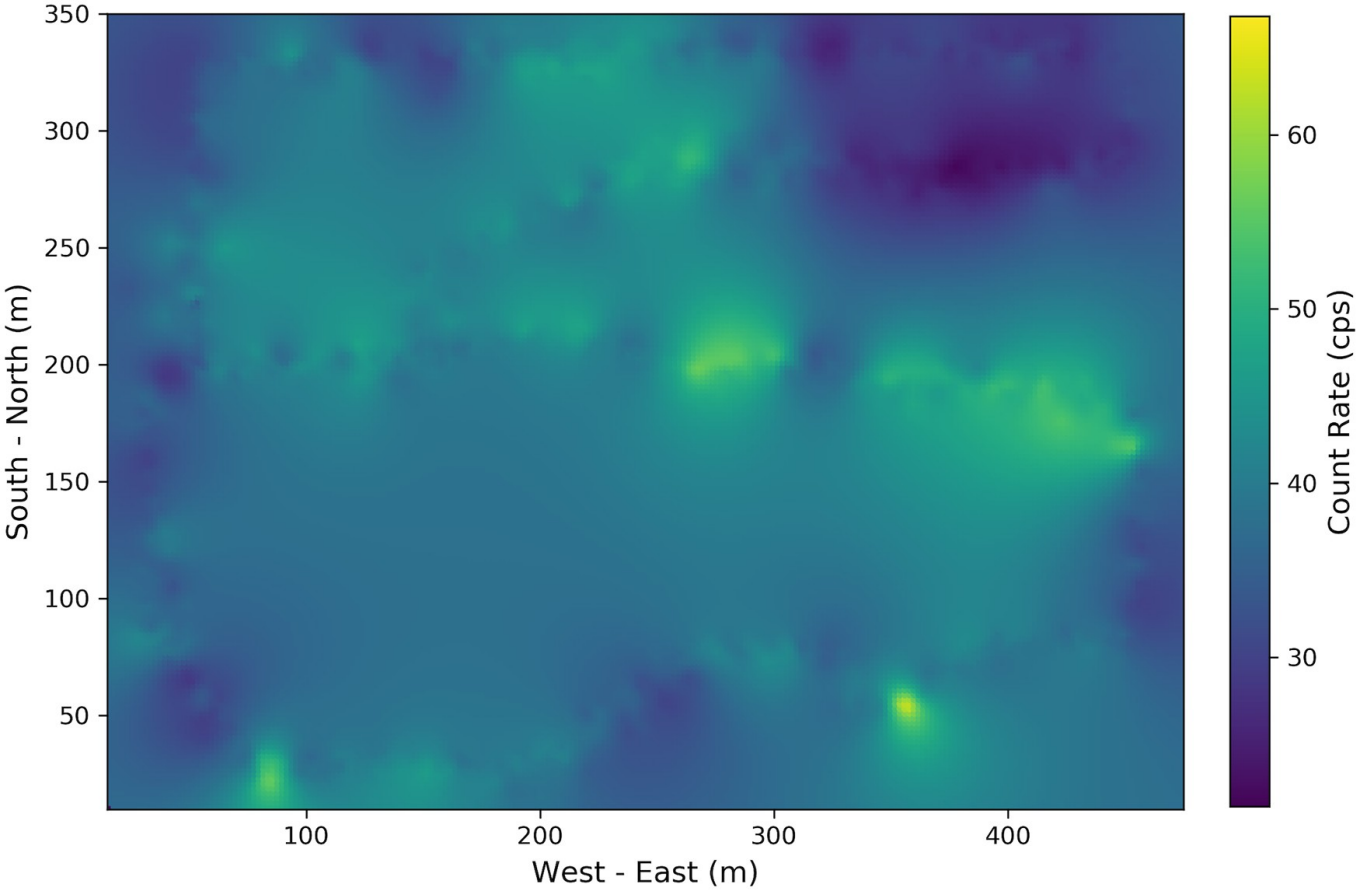
Poisson kriging estimation of background radiation distribution.

Based on the fitted semi-variogram from Fig 3 and the background radiation measurements shown in Fig 1, the Poisson kriging was used to produce the interpolation kernel density surface. The result is illustrated in Fig 4, which provides the smooth distribution of background radiation levels. The lighter color (e.g., yellow color) represents a greater radiation level.

### Anomalous source detection

The goal of the research work is not only to estimate background radiation levels based on the radiation data collected using mobile sensor networks, but also to identify the location of the anomalous radiation source. In real cases, two types of data are available: the historical background radiation data where no radioactive sources are present and the newly collected data which might contain radioactive sources. To identify the anomalous radioactive sources, this work implements a two-step method. First, the distribution of the background radiation of the study area is estimated using Poisson kriging model. In the second step, we inserted the radioactive sources in the study area and the newly collected data will contain the information of the radioactive sources, from which, the anomaly score can be defined to identify the radioactive sources.

More specifically, for position ***r***, using the historical background radiation data, the estimated background radiation level is denoted by $Y_{r}^{b}$. After suspicious data are collected, the estimated radiation level from Poisson kriging based on the newly measured data is denoted by $Y_{r}^{n}$. The difference between the estimation from newly measurements and from background data is used to define the anomaly score:

$$\begin{array}{c} Score\left(Y_{r}^{n};Y_{r}^{b}\right)=Y_{r}^{n}-Y_{r}^{b}. \end{array}$$ (18)

Eq 18 simply uses the difference between new estimation and background radiation estimation to define anomaly score. It works when the background radiation distribution is smooth. However, in real cases, the high fluctuation areas always exist. Therefore, a more robust anomaly score needs to be developed based on the SNR. In SNR, the square root of background radiation level is used as the estimation of noise level, and the difference between new estimation and background radiation level is used as the estimation of signal strength. The anomaly score is then defined as:

$$\begin{array}{c} Score\left(Y_{r}^{n};Y_{r}^{b}\right)=\frac{Y_{r}^{n}-Y_{r}^{b}}{\sqrt{Y_{r}^{b}}}. \end{array}$$ (19)

**Fig 5:**
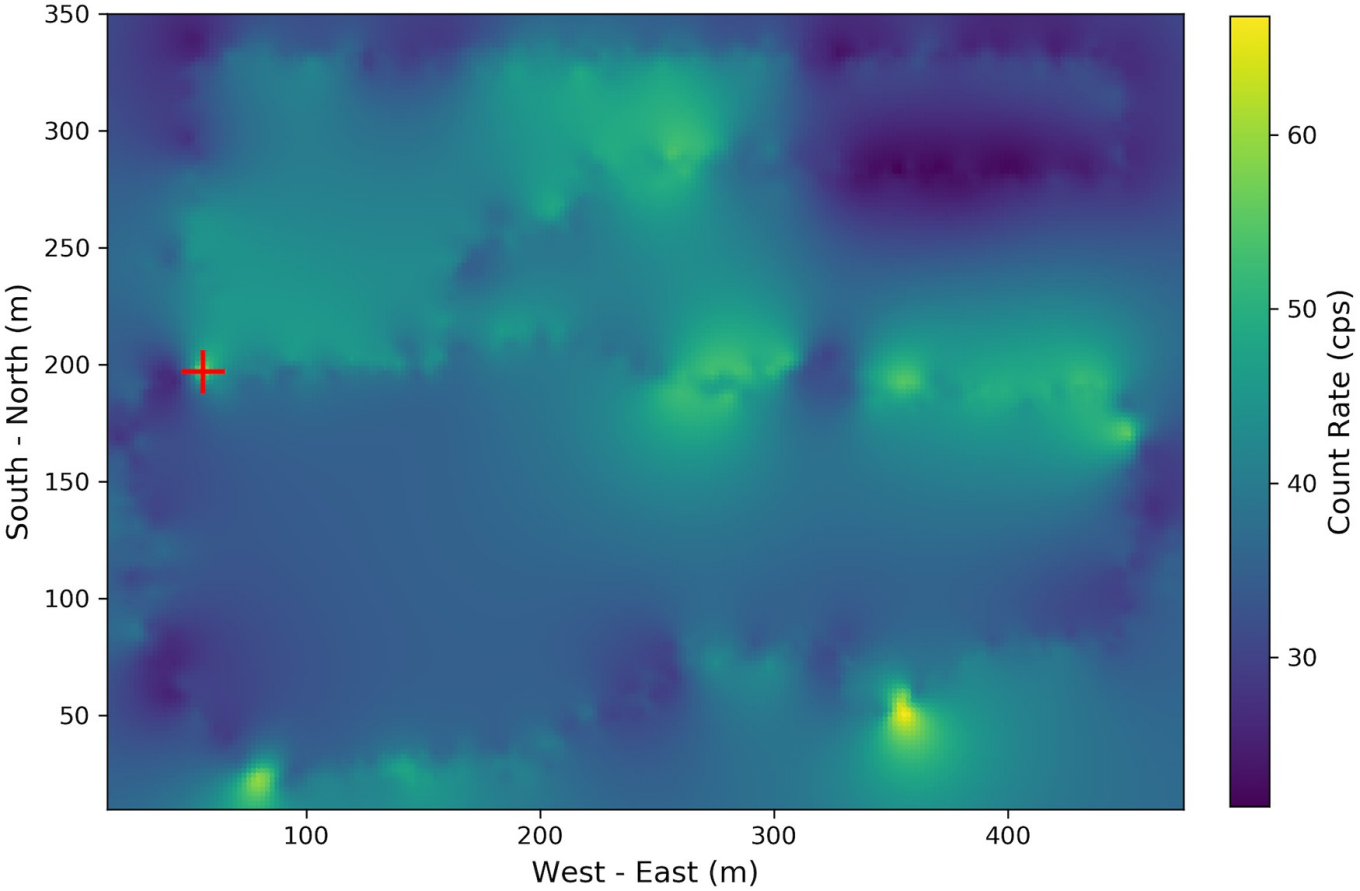
Poisson kriging estimation with synthetic source injection. The 100 *μCi*^137^*Cs* source is injected 1 meter away from the walking path, which is denoted using a red cross.

To test the proposed method, considering the case where a simulated radioactive source with 100 *μCi* source intensity is injected 1 meter away from walking paths as denoted by number 1 in Fig 1. After adjusting the collected data as described in Fig 2, Poisson kriging is applied and the estimated radiation distribution is shown in Fig 5, where the injected radioactive source is denoted by the red cross.

**Fig 6:**
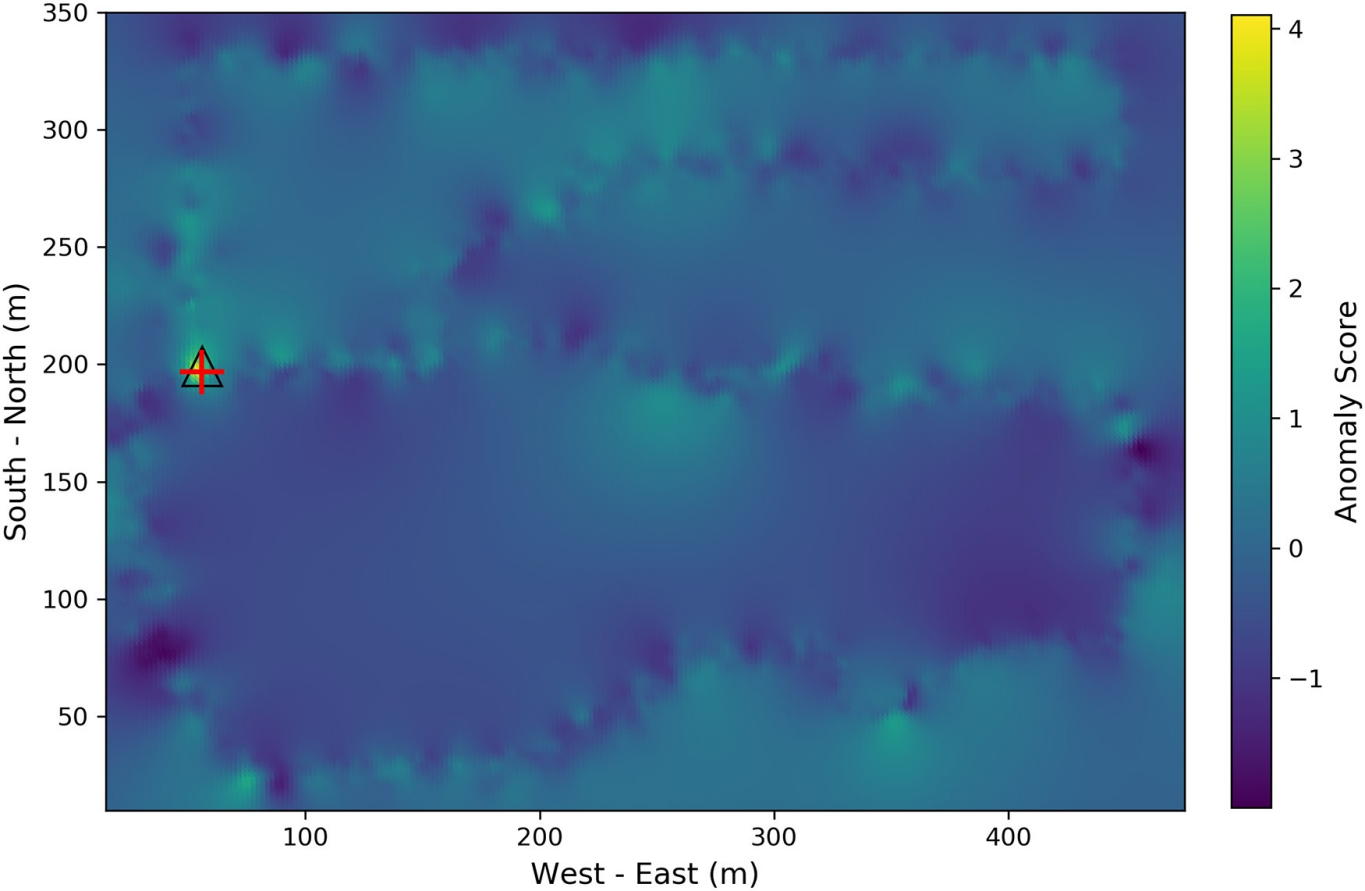
Anomalous source localization from Poisson kriging model. The background color corresponds to the anomaly score calculated using Eq 19. The injected source was denoted by red cross and the estimated source location was denoted by black triangle.

Compared with Fig 4, the significant difference appears at the location where the simulated source location is. However, in Fig 5, although the hot spot area around the injected source is identified, the high background radiation regions make it hard to correctly identify the radioactive source purely based on the difference between Fig 5 and Fig 4. To make more robust decisions, the anomaly score defined in Eq 19 is computed and the distribution of the anomaly score is shown in Fig 6. The actual source location is denoted by the red cross and the estimated source location based on maximal anomaly score is denoted by the black triangle. Through calculating SNR, the influence of high background radiation region is mostly eliminated, and the radioactive source is correctly located.

### Error analysis

Previous sections demonstrates the application of Poisson kriging for nuclear radiation distribution estimation and anomalous radioactive source identification using an example case. Here, we developed comprehensive test cases to evaluate the performance of the model. The ^137^*Cs* source with 5 different intensities (100 *μCi*, 300 *μCi*, 500 *μCi*, 1,000 *μCi*, and 2,000 *μCi*) was added to 10 randomly selected locations in the study area as shown in Fig 1. The distance to the walking paths varied from 1 m to 10 m. We choose the model’s source identification accuracy to evaluate its performance. The source identification accuracy is calculated according to the given distance threshold. When the distance of the estimated source location from the actual source location is smaller than the distance threshold, the source is assumed to be correctly identified. In this study, we defined three distance threshold values (5, 10, and 20 meters). For each distance threshold scenario, we calculated the source identification accuracy. The results are illustrated in Tables 3, 4, and 5.

**Table 3:** Source identification accuracy with 5 meters distance threshold.

Configuration		Distance to the Walking Path		
		1 m	5 m	10 m
Source Intensity	100 *μCi* (3.7 × 10^6^ Bq)	80%	0%	0%
	300 *μCi* (1.11 × 10^7^ Bq)	100%	40%	0%
	500 *μCi* (1.85 × 10^7^ Bq)	100%	50%	20%
	1,000 *μCi* (3.7 × 10^7^ Bq)	100%	60%	30%
	2,000 *μCi* (7.4 × 10^7^ Bq)	100%	70%	30%

**Table 4:** Source identification accuracy with 10 meters distance threshold.

Configuration		Distance to the Walking Path		
		1 m	5 m	10 m
Source Intensity	100 *μCi* (3.7 × 10^6^ Bq)	90%	20%	10%
	300 *μCi* (1.11 × 10^7^ Bq)	100%	90%	20%
	500 *μCi* (1.85 × 10^7^ Bq)	100%	100%	40%
	1,000 *μCi* (3.7 × 10^7^ Bq)	100%	100%	50%
	2,000 *μCi* (7.4 × 10^7^ Bq)	100%	100%	60%

**Table 5:** Source identification accuracy with 20 meters distance threshold.

Configuration		Distance to the Walking Path		
		1 m	5 m	10 m
Source Intensity	100 *μCi* (3.7 × 10^6^ Bq)	90%	20%	10%
	300 *μCi* (1.11 × 10^7^ Bq)	100%	90%	20%
	500 *μCi* (1.85 × 10^7^ Bq)	100%	100%	50%
	1,000 *μCi* (3.7 × 10^7^ Bq)	100%	100%	90%
	2,000 *μCi* (7.4 × 10^7^ Bq)	100%	100%	100%

As expected, the performance of the proposed method is dramatically influenced by source intensity and the distance of radioactive source from the walking paths. When the source is close enough or the source intensity is high enough or both, the identification accuracy could reach 100%. However, when the source intensity is low or the distance of radioactive source from the walking paths is too large, radioactive sources cannot be easily identified.

### Discussion

In this work, Poisson kriging is not only used for background radiation levels estimation, but also for radioactive source identification. The performance of the proposed framework is analyzed using a small mobile sensor network and experimental data with simulated radioactive source. The results indicate that the proposed algorithm can find the radioactive source with high accuracy if the source is close enough to the walking paths (e.g., less than 5 meters away) or if the radioactive source is strong enough (e.g., 500 *μCi* or above). For cases where the radioactive source is placed 5 meters away from the walking paths, the number of successful cases increases rapidly when the source intensity increases. The model yields poor performance when the source is placed more than 10 meters away from the walking paths. In these cases, the local hot spot areas around the radioactive sources can always be identified, which provide important prior information for further investigation.

On the other hand, there are several uncertainty factors involved in this study, which might cause the error of the results. The radiation level that is measured using a mobile sensor is significantly affected by the weather condition, detector’s shielding condition, and moving speed. The correlation between those factors and the background radiation level is not analyzed in this work. Also, the current Poisson kriging model assumes that the background radiation level has the same mean value among the study area. A more advanced hierarchical model [23] can also be used to avoid the uniform mean assumptions.

The main focus of the article is to develop a method that can be used to detect anomalous radioactive source. In this article, the proposed method was tested using a small mobile sensor network. The data collection and transmission processes were done in near real-time. In the future, the computational complexity should be considered and the optimal threshold for anomalous source identification should be determined using more experimental data. Also, this algorithm should be implemented in a way that it can run parallel processes and perform big geospatial data computing at cloud in real-time.

## Conclusion

The goal of the project is to detect anomalous radioactive sources (e.g., nuclear bombs or weapons) using mobile sensor networks. There are several innovations involved in this research work. The data streams that are collected through continuous interaction between time and space require real-time (or near real-time) analytics and response. In contrast, most spatial analysis methods and computing framework have not pursued this goal, thus reducing their effectiveness in decision support contexts and motivating research conducted to improve the performance of data-intensive geospatial analysis. In this research work, we developed an intelligent mobile sensor network, in which the radiation streaming data collected using mobile sensors in every second are automatically transferred to the cloud in the form of geo-tagged streaming data in near real-time. In the second step, we developed a novel spatial algorithm based on Poisson kriging to detect the anomalous radiation source. We have conducted experiments with simulated radioactive sources to test the proposed method’s performance. The results indicate that the proposed algorithm can correctly capture the spatial distribution of nuclear radiation levels and find the anomalous radiation source with extremely high accuracy under certain conditions.
